# G418 induces programmed cell death in *Acanthamoeba* through the elevation of intracellular calcium and cytochrome *c* translocation

**DOI:** 10.1007/s00436-018-6192-0

**Published:** 2019-01-07

**Authors:** Zisis Koutsogiannis, Ewan T. MacLeod, Sutherland K. Maciver

**Affiliations:** 1 Centre for Discovery Brain Sciences, Edinburgh, UK; 2 Division of Infection and Pathway Medicine, Biomedical Sciences, Edinburgh Medical School, University of Edinburgh, Hugh Robson Building, George Square, Edinburgh, Scotland EH8 9XD UK

**Keywords:** *Acanthamoeba*, Amoebozoa, Programmed cell death, Apoptosis, G418, Cytochrome *c*

## Abstract

*Acanthamoeba* is a widely distributed opportunistic parasite which causes a vision-threatening keratitis and a life-threatening encephalitis. The cyst stage of this amoeba is especially resistant to currently used therapeutics and so alternative agents are urgently required. Growing evidence supports the existence of a programmed cell death system (PCD) in *Acanthamoeba* and while some features are shared by higher eukaryote cells, others differ. It is hoped that by understanding these differences we can exploit them as targets for novel drug intervention to activate PCD pathways in the amoebae but not the invaded human tissue. Here, we use the aminoglycoside G418 to activate PCD in *Acanthamoeba*. This drug caused a shape change in the treated amoebae. Cells rounded up and contracted, and after 6 h fragments of cells resembling the ‘apoptotic bodies’ of vertebrate cells were observed. G418 causes an increase in intracellular calcium from a resting level of 24 nM to 60 nM after 6 h of treatment. Mitochondrial function as assayed by the ΔΨ_m_ reporting dye JC-1 and CTC a redox dye becomes inhibited during treatment and we have found that cytochrome *c* is released from the mitochondria. Cells stained with Hoechst showed first an alteration in chromatin structure and then a vesiculation of the nucleus with G418 treatment, although we found no obvious breakdown in genomic DNA in the early stages of PCD.

**Electronic supplementary material:**

The online version of this article (10.1007/s00436-018-6192-0) contains supplementary material, which is available to authorized users.

## Introduction

*Acanthamoeba* is a eukaryotic, opportunistic parasitic protozoan, which is widely distributed in the natural environment (Geisen et al. 2014). Although species classification has not been completely formalized, the genus has been divided into 21 different genotypes, T1-T21 (Stothard et al. 1998; Booton et al. 2002; Corsaro and Venditti 2018) based on 18S rRNA gene sequences which equates approximately to classification at the species level (Hewett et al. 2003; Fuerst 2014). While some named species accord with this system, others do not. The most commonly encountered genotype is T4, and while it is the most frequently isolated from the environment, it is also disproportionally associated with pathology (Maciver et al. 2013). *Acanthamoeba* (especially T4) is the causative agent of *Acanthamoeba* keratitis (Lorenzo-Morales et al. 2015) a painful, vision-threatening infection and worse, a granulomatous amoebic encephalitis (GAE), an infection of the central nervous system which is usually fatal (Duggal et al. 2017). Many drugs have been used to combat *Acanthamoeba* infections (Siddiqui and Khan 2012; Martín-Navarro et al. 2013; Lorenzo-Morales et al. 2016), but they tend not to be as effective due to a lack *Acanthamoeba* specificity and some commonly used drugs are known to cause harm to the patient at currently used dosages (Ehlers and Hjortdal 2004; Moon et al. 2018). Part of the problem is that *Acanthamoeba* alternates between the active and infective trophozoite, and a dormant, double walled cyst. Treatment of the infections caused by *Acanthamoeba* is made difficult by the presence of these cysts because they are resistant to many disinfectants and other treatments (Lorenzo-Morales et al. 2015). New drugs are urgently needed, but *Acanthamoeba* is a eukaryote which means that its biochemical pathways are unfortunately similar to that of their human hosts, limiting the availability of specific drug targets. It is clear that we need to identify and exploit differences between *Acanthamoeba* and ourselves to find better drug targets, and we argue that such differences may be found within the programmed cell death (PCD) system.

It was something of a surprise to find that vertebrate cells possess the deliberate means of self-destruction (Kerr et al. 1972) as this seems so blatantly anti-Darwinian, but we now know that cells are required to eliminate themselves in a variety of ways in several different situations within the vertebrate body especially during development. The suggestion that single celled organisms can also kill themselves is even more unexpected, but evidence is accumulating to support this. PCD processes have been identified and described in numerous unicellular organisms including yeast such as *Saccharomyces* (Ludovico et al. 2001; Madeo et al. 1999) slime molds such as *Dictyostelium* (Cornillon et al. 1994), parasitic protozoans including *Trypanosoma* (Welburn et al. 1996; Nguewa et al. 2004; Duszenko et al. 2006; Jiménez-Ruiz et al. 2010)*, Leishmania* (Lee et al. 2002), *Entamoeba* (Villalba et al. 2007) and *Plasmodium* (Al-Olayan et al. 2002). Although details on the actors of PCD are scant for protists as they generally lack the caspase enzymes that are commonly seen in vertebrates. For example, caspases are absent in *Dictyostelium discoideum* (Olie et al. 1998), *Acanthamoeba castellanii* (Clarke et al. 2013) and *Naegleria gruberi* (Fritz-Laylin et al. 2010). It is hoped that a fuller understanding of these PCD pathways will reveal targets that may act as efficient ways of killing these parasites through drugs designed to activate parasite but not host PCD pathways. The aim of this study is to investigate the role of calcium influx and mitochondrial misfunction in the early stages of the process of G418-induced programmed cell death in *Acanthamoeba*.

## Materials and methods

### Amoebae isolation, culture and microscopy

A new strain of *Acanthamoeba*, GS-336, was isolated from a soil sample taken from George Square gardens, Edinburgh, using the modified (De Obeso Fernandez Del Valle et al. 2017) ‘walkout’ method of Neff (Neff 1958). Soil was dissolved in Neff’s saline buffer NSB see below), placed on a non-nutrition 2.0% agar plate (2% agar NSB) and left overnight at room temperature. Small sections of agar were excised, inverted and placed on a second-fresh non-nutrition 2.0% agar plate onto which *Eschericia coli* bacteria have been spread. This was repeated until a pure population of *Acanthamoeba* was established. Clonal strains were then adapted to axenic media (see below) and their T-type determined by PCR by the method of Lorenzo-Morales (Lorenzo-Morales et al. 2005). One particular T4 strain, GS-336, that was closely related to the reference *Acanthamoeba castellanii* Neff strain (ATTC30010, CCAP1501/1B) and was used in this study and routinely cultured in axenic media (7.15 g/l yeast extract, 14.3 g/l peptone, 15.4 g/l glucose, 0.486 g/l KH_2_PO4, 0.51 g/l Na_2_HPO4, pH 6.5). Experiments were conducted in Neff’s saline buffer (NSB) (NaCl 0.12 g/L, MgSO_4_7H_2_O 4 mg/L, CaCl_2_ 4 mg/L, Na_2_HPO_4_ 0.142 g/L, KH_2_PO_4_ 0.136 g/L). Light microscopic observations were made on living amoebae using an inverted Leica DMIRB microscope equipped with Hoffman modulation contrast optics. Imaging/image analysis was performed in the IMPACT Imaging Facility, Centre for Discovery Brain Sciences, University of Edinburgh. Confocal microscopy was carried out using a Nikon A1R microscope with various filters. NIS Elements software was used for all collected images which were then further processed with Imaris and ImageJ software.

### Phylogenetic analysis

Sequences were compiled with others from Genbank and aligned using the ‘muscle’ algorithm (Edgar 2004) implemented using ‘Seaview’ version 4 (Gouy et al. 2010). The alignments were then manually trimmed using ‘BioEdit’ (Hall 1999) and maximum likelihood phylogenetic trees produced by PhlyML algorithm with the GTR model (Guindon and Gascuel 2003). The non-parametric analysis was performed with 1000 bootstrap pseudo-replicates, using the 18S sequence from *Acanthamoeba pyriformis* (Tice et al. 2016) as the outgroup.

### Viability assays

Viability of trophozoites was determined in the presence or absence of the aminoglycoside G418 for different periods of time by trypan blue exclusion assay. *Acanthamoeba* cells were suspended in 0.4% trypan blue dye in NSB and left for 5 min at room temperature. The percentage of cell viability was measured as:

$$ \frac{\mathrm{Total}\ \mathrm{viable}\ \mathrm{cells}}{\mathrm{Total}\ \mathrm{number}\ \mathrm{of}\ \mathrm{cells}}\times 100\% $$

Cells were counted manually using a haemocytometer and an inverted microscope (× 20 objective).

### Intracellular Ca^2+^ concentration

Intracellular levels of calcium were monitored by the fluorescent probe Fura-2/AM. After treatment for various periods with G418 (IC_90_ concentration) *Acanthamoeba* trophozoites were harvested and washed twice in Neff’s saline buffer at 500×*g* for 5 min at room temperature. Trophozoites were resuspended in Fura 2/AM loading buffer containing: 6 μM Fura 2 AM, 116 mM NaCl, 54 mM KCl, 0.8 mM MgSO_4_, 55 mM D glucose, 0.05 M HEPES, pH 7.4 (Villalba et al. 2007) for 30 min at 37 °C. Afterwards, trophozoites were washed twice with 1 mL NSB twice to remove dye excess. Fura 2 AM was excited at 340/380 nm with 510 nm emission in Perkins-Elmer fluorimeter. The [Ca^2+^]_i_ was determined at room temperature using the following equation:

$$ \left[{Ca}^{2+}\right]i={K}_d\times \beta \times \frac{\left[R-R\min \right]}{\left[R\max -R\right]} $$

*R* = sample ratio fluorescence, *R*_min_ = calcium-zero conditions (EGTA 10 μΜ), *R*_max_ = calcium-saturated conditions (CaCl_2_ 4 mM + 10 μM ionomycin) and *β* = ratio of *R*min/*R*max at 380 nm, *K*_d_ = 224 nM at 30 °C (Grynkiewicz et al. 1985).

### Hoechst 33342 staining protocol and Hoechst fluorescence intensity

Hoechst 33342 is a cell-permanent nucleic acid stain which we have used to study chromatin structure in *Acanthamoeba*. Briefly, trophozoites treated with IC_90_ G-418 in NSB and incubated at 37 °C for preschedule period (0 to 8 h). Amoebae were then centrifuged at 500×*g* for 10 min, washed once with NSB and fixed with fresh 4% paraformaldehyde (PFA) for 20 min at room temperature. Cells washed again with NSB and centrifuged at 500×*g* for 10 min. Hoechst 33342 was applied at a final concentration of 10 μM. Cells incubated at 37 °C in the dark for 30 min and finally washed twice with 1 mL NSB to remove dye excess. Stained trophozoites were observed by confocal microscopy, under an epifluorescence microscope (Nikon A1R). DAPI filters were applied in order to detect fluorescence. Analysis was later made using Image analysis Software Imaris and ImageJ. Emission signal intensity of Hoechst 33342 was monitored and measured by a LS55 PerkinElmer luminesce spectrometer when excited at 510 nm.

### CTC staining and fluorescence intensity

CTC (5-cyano-2,3-ditolyl tetrazolium chloride) is a redox dye used here to report the respiratory activity of *Acanthamoeba* mitochondria. Respiring *Acanthamoeba* trophozoites loaded with CTC solution reduce the dye to produce a visible insoluble formazan, conversely, dead or mitochondrial dysfunctional trophozoites show lower or no such accumulation. *Acanthamoeba* were treated with IC_90_ G418 in NSB and incubated at 37 °C for various periods (1–3–6 h). Cells were centrifuged at 500×*g* for 10 min. Cells washed twice with NSB and centrifuged as before. 5-Cyano-2,3-ditolyl tetrazolium chloride at a final concentration of 5 mM in NSB were applied and cells incubated at 37 °C, in dark for 30 min before washed twice with 1 mL NSB to rinse dye excess. Cells were fixed with 4% paraformaldehyde for 20 min, washed with 1 mL NSB and centrifuged at 500×*g* for 10 min before applied on a micro slide and observed by confocal microscopy, Epi-Filter rhodamine red; excitation/emission (nm): 480/630 using NIS elements software. Fluorescence was quantified using a LS55 Perkin Elmer luminesce spectrometer.

### Mitochondrial membrane potential measurement

Mitochondrial membrane potential was measured using the florescent reported, JC-1. This membrane permeant dye accumulates in mitochondria at a rate determined by the potential difference across the mitochondrial membrane. The monomer emits around 530 nm and as it concentrates in mitochondria it forms fluorescent aggregates which emit a longer wavelength centred at 590 nm. Consequently, a reduction in the J-aggregate fluorescence indicates depolarization whereas a rise means hyperpolarization. Trophozoites were treated with G418 IC_90_ for predefined periods (1–3–6 h), harvested and washed twice with NSB. After centrifuging at 500×*g* cells were suspended in Neff’s saline buffer containing 6 μM JC-1 and left for 20 min at 37 °C, in dark. Trophozoites were then washed twice with 1 mL NSB and centrifuged. Cells were examined immediately by fluorescence microscopy using excitation FITC (490-540 nm) and emission JC-1 (580–610 nm) filters. Simultaneously, JC-1 fluorescence was measured spectrophotometrically at 488 nm using a LS55 Perkin Elmer luminesce spectrometer.

### Release of cytochrome *c* from mitochondria

Cytochrome *c* release was assayed using a commercial kit (Abcam’s cytochrome *c* releasing apoptosis assay kit (ab65311)). *Acanthamoeba* trophozoites were incubated with IC_90_ G418 in NSB at 37 °C for predetermined period (0, 1, 3, 6 h). Cells were collected in Eppendorf tubes and centrifuged at 500*g* for 5 min, washed once with NSB and supernatant was removed. One millilitre of 1x cytosol extraction buffer mix containing DTT and protease inhibitors was used to re-suspend the pellet while sample was incubated in ice for 10 min. Approximately 4–6 cycles of freeze–thaw were conducted using dry ice to freeze *Acanthamoeba* suspension (~ 4 min) and a tube thermal incubator set at 37 °C (~ 3 min). The homogenization process was monitored under a microscope by applying 5–6 μL of cell suspension onto a coverslip. The homogenate was centrifuged at 10,000*g* to separate cytosolic from mitochondrial fraction for 30 min at 4 °C. Supernatant was collected in a new Eppendorf tube and labeled as cytosolic fraction. Pellet was re-suspended in 100 μL mitochondrial extraction buffer mix that contained DTT and protease inhibitors, while was vortexed for 10 to 15 s and labeled as mitochondrial fraction. Protein concentrations were determined using a Pierce BCA protein assay kit. Samples (10 μg) of each cytosolic and mitochondrial fractions isolated from treated and untreated *Acanthamoeba* were separated by 16% SDS-PAGE and then Western Blotted using a Bio-Rad ‘mini protean’ system and probed with a polyclonal rabbit anti-cytochrome *c* antibody (Abcam ab90529) at a final dilution of 1:200 in skimmed dry milk 3.5% *w*/*v*, in tris-buffered saline (TBS). 0.4 mm PVDF membranes were incubated at 4 °C overnight with slight agitation and washed twice for 5 min with TBS-T (TBS-0.1% Tween-20). A secondary fluorescent antibody was used to label rabbit’s anti-cytochrome *c* antibody. Odyssey goat anti-rabbit IgG IR dye was diluted in skimmed dry milk 3.5% *w*/*v*, in tris-buffered saline to 1 μg/mL and applied to PVDF protein membranes for 1 h at room temperature, in the dark with gentle agitation. Membrane were washed twice for 5 min with TBS-T at room temperature, visualized with a LI-COR Odyssey Classic imager scanner and analyzed by win Image studio 5.2 analysis software.

## Results

The strain of *Acanthamoeba* used for this study GS-336 was chosen as it was similar to the well-characterised T4 Neff strain (*Acanthamoeba castellanii* ATCC 30010, CCAP1501/1A) whose genome has been sequenced (Clarke et al. 2013). It was important to use a fresh strain since the original Neff strain has become altered by its prolonged period in culture (Köhsler et al. 2008). Phylogenetic analysis shows that GS-336 is a T4 strain and groups with the Neff strain (Supplementary Fig. 1).

### G418 effect on Acanthamoeba trophozoites viability and morphology

**Fig. 1:**
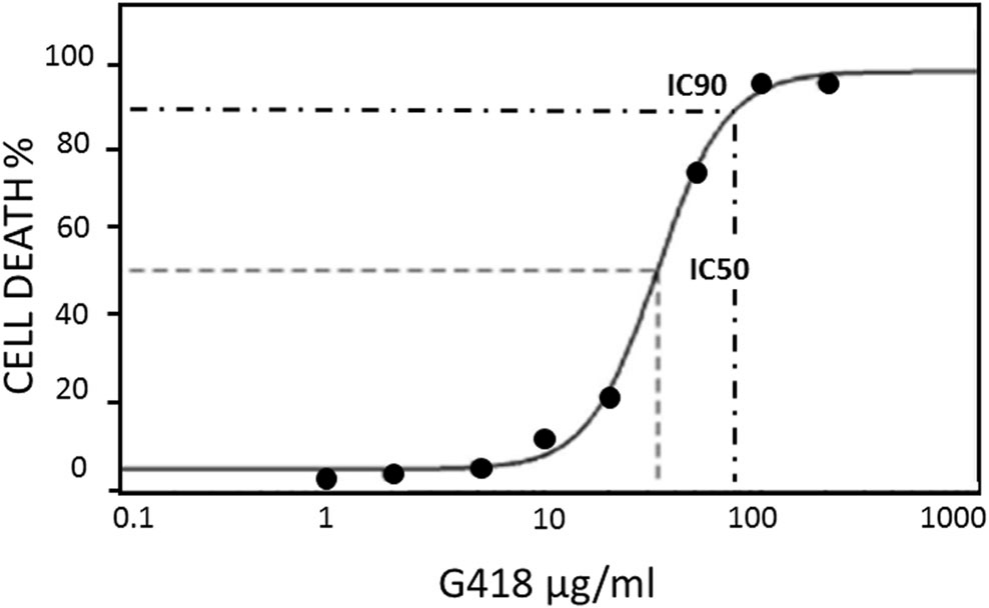
The killing of *Acanthamoeba* by a range of concentrations of G418 established that the IC_50_ value is 32 ± 3 μg/mL while the IC_90_ value is 75 ± 5 μg/mL. Cells were incubated with G418 in NSB for 24 h at 37 °C prior to determining cell death by trypan blue exclusion

**Fig. 2:**
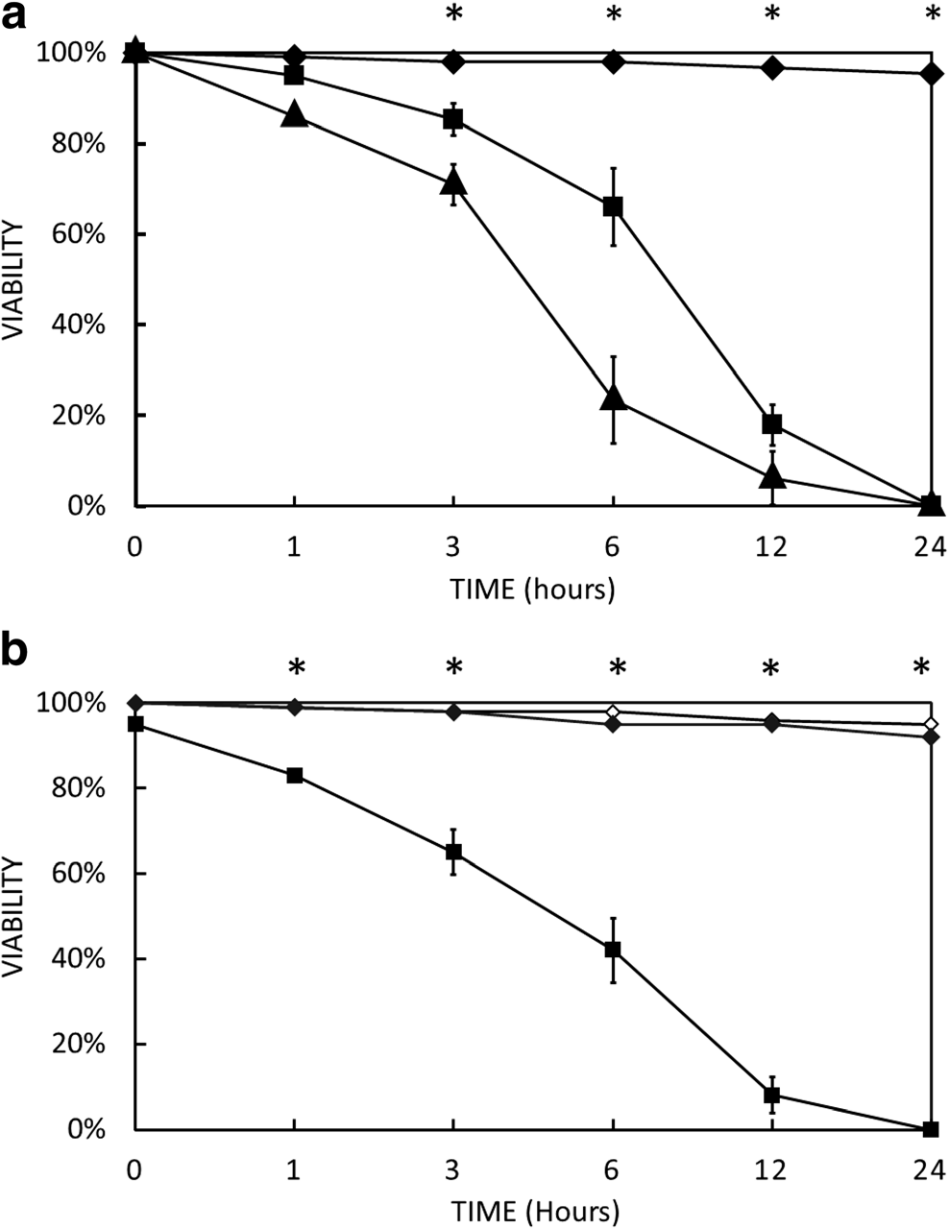
Viability of *Acanthamoeba* trophozoites measured by trypan blue exclusion as a function of time. Errors bars represent S.E. Significance is indicated (*) *P* < 0.05, *t* test, two-tailed. **A**. Cell were treated with 0 (black diamond), 100 (black square), and 200 (black up-pointing triangle) μg/mL of G418 at 37 °C in NSB. **B**. Cells treated with 100 μg/mL of G418 for 1 h then washed in NSB at 37 °C (black square). Cells treated with 100 μg/mL of G418 at room temperature (black diamond). Control cells with no G418 (white diamond)

**Fig. 3:**
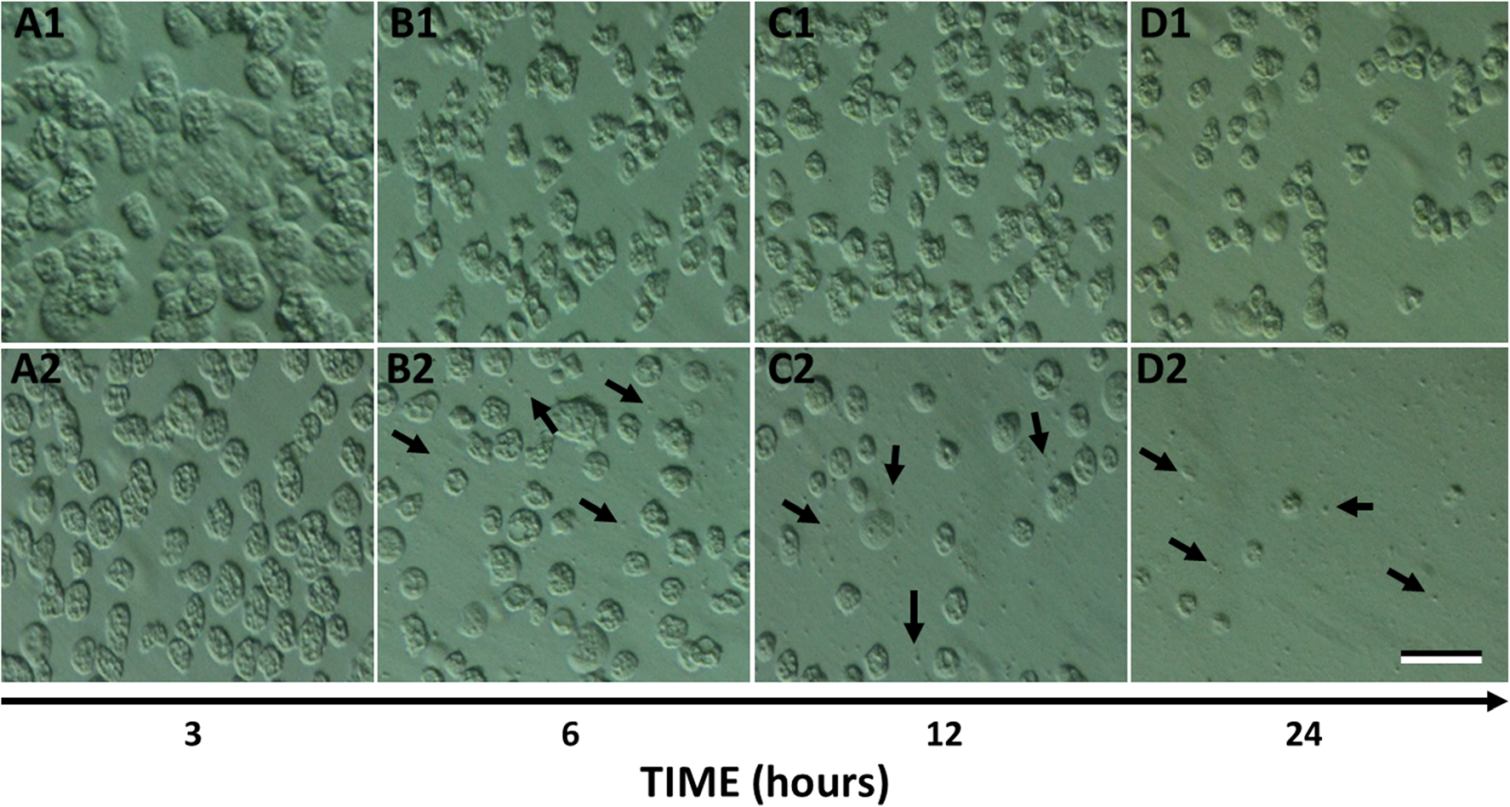
Morphology of untreated (A1–D1) and IC_90_ G418 treated (A2–D2) *Acanthamoeba* trophozoites during time under light microscopy. Black arrows indicate small particles which resemble ‘apoptotic bodies’. Images are representative of three independent experiments. Scale bar represents 50 μM

In agreement with earlier work (Peng et al. 2005) the aminoglycoside G418 was found to kill *Acanthamoeba* trophozoites. We have established that the IC_50_ and IC_90_ values at 37 °C are 32 ± 3 and 75 ± 5 μg/mL respectively in NSB (Fig. 1). The full amoebicidal effect is only apparent around 24 h and is temperature-dependent (Fig. 2B). After 1 h of treatment with G418 (100 μg/mL) at 37 °C, viability remained at 95% while the control amoebae showed only very minor differences. The viability in treated amoebae remained high even after 3 h when only 12% of the trophozoites were determined to be dead. However, G418 treated cells were seen to be more ‘rounded up’ compared to control amoebae which maintained their spread and adherent morphology (Fig. 3A_1_–D_2_). After 6 h with G418 treatment, trophozoite viability dropped to 66%, and more of the amoebae were spherical, whereas control amoebae were unaffected. At this time, a small number of sub-cellular-sized particles became visible (Fig. 3B_2_). After 12 h of G418 treatment *Acanthamoeba* viability diminished to 18%, cell density was greatly decreased, and particulate material was more numerous (Fig. 3C_1_). Control amoebae preserved their high viability rates up to 97%; however, they had become more rounded up possibly due to incubation in the absence of glucose (Fig. 3C_2_). The entire population of *Acanthamoeba* trophozoites treated with 100 μg/mL G418 had completely died after 24 h of incubation at 37 °C and there were also obvious signs of complete cell lysis (Fig. 3D_2_). Higher G418 concentration led also to same but prompter and devastating results. Interestingly, identical treatment with G418 in NSB at room temperature failed to mediate cell death in *Acanthamoeba*, making temperature stress a precondition to cell death induction. In addition, G418 is able to cause cell death in trophozoites cells even after its removal after an hour which is an indication that it is an initiator of cell death rather than the executor (Fig. 2B).

### G418 increases intracellular calcium concentration

**Fig. 4:**
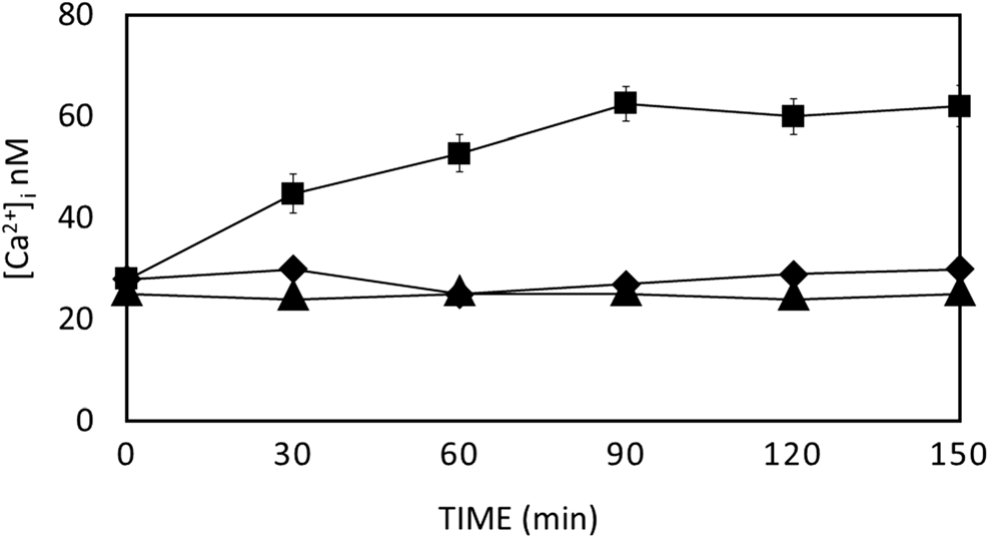
Intracellular calcium levels in G418-treated (IC_90_) *Acanthamoeba* trophozoites (black square) and control amoebae (black diamond) (no G418). Negative control column represents treated amoebae after the addition of 10 μΜ EGTA (black up-pointing triangle), errors bars represent S.E., *P* < 0.05, *t* test, two-tailed

Spectrophotometric analysis of Fura-2-loaded amoebae revealed that the levels of intracellular calcium was higher in the trophozoites treated with the aminoglycoside in a period of 150 min while at the same time the amount of intracellular calcium in the control population remained stable. During the first 90 min of incubation, [Ca^2+^]_i_ showed a rising trend ranging from 42 ± 3.8 nM at 30 min, 54 ± 4.2 at 60 min, with a maximum of 62 ± 4.5 nM at 90 min, (*P* < 0.05). CaCl_2_/ Ionomycin and EGTA was used as positive and negative controls respectively and as expected significantly increased and diminished the intracellular concentration of calcium in both controlled and treated trophozoites (Fig. 4).

### G418 induces nuclear morphological changes

**Fig. 5:**
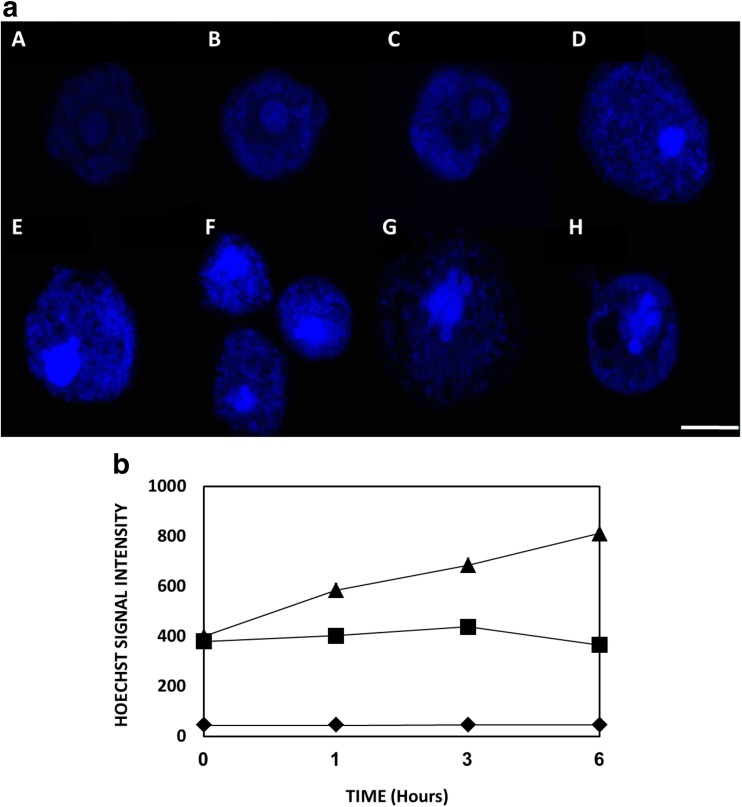
**a** Images A to H show a 1-h time series highlighting the nuclear morphology of *Acanthamoeba* trophozoites after treatment with IC_90_ G418 and stained with Hoechst 33342. Images are representative of three independent experiments. Scale bar 5 μM. **b** Hoechst 33342 fluorescence intensities of IC_90_ G418 treated (black triangle) and untreated (black square) cells at 1, 3 and 6 h. Unlabeled cells (black diamond)

Hoechst 33342 staining (Fig. 5a) revealed developing changes in the morphology of the nuclei of G418 treated *Acanthamoeba* trophozoites compared to the control population. These changes occur over a period of between 4 and 8 h which is typical of programmed cell death mechanisms in other systems. The first signs, visible after 4 to 6 h of incubation, where nuclear shrinking and simultaneously the appearance of punctate foci of more intensely staining sub-nuclear regions (Fig. 5a). After 8 h, nuclear breakdown was evident where lobes of chromatin spilled out of the nucleus into the cytoplasm indicating that the nuclear envelope had broken down, while the general staining continued to increase in intensity (Fig. 5A part g) consistent with continued chromatin condensation. This increase in Hoechst staining intensity was quantified spectrophotometrically (Fig. 5B). However, despite the obvious changes to the nucleus, no change was evident in the appearance of genomic DNA when isolated and run on agarose gels (data not shown).

### Mitochondria in *Acanthamoeba* PCD

**Fig. 6:**
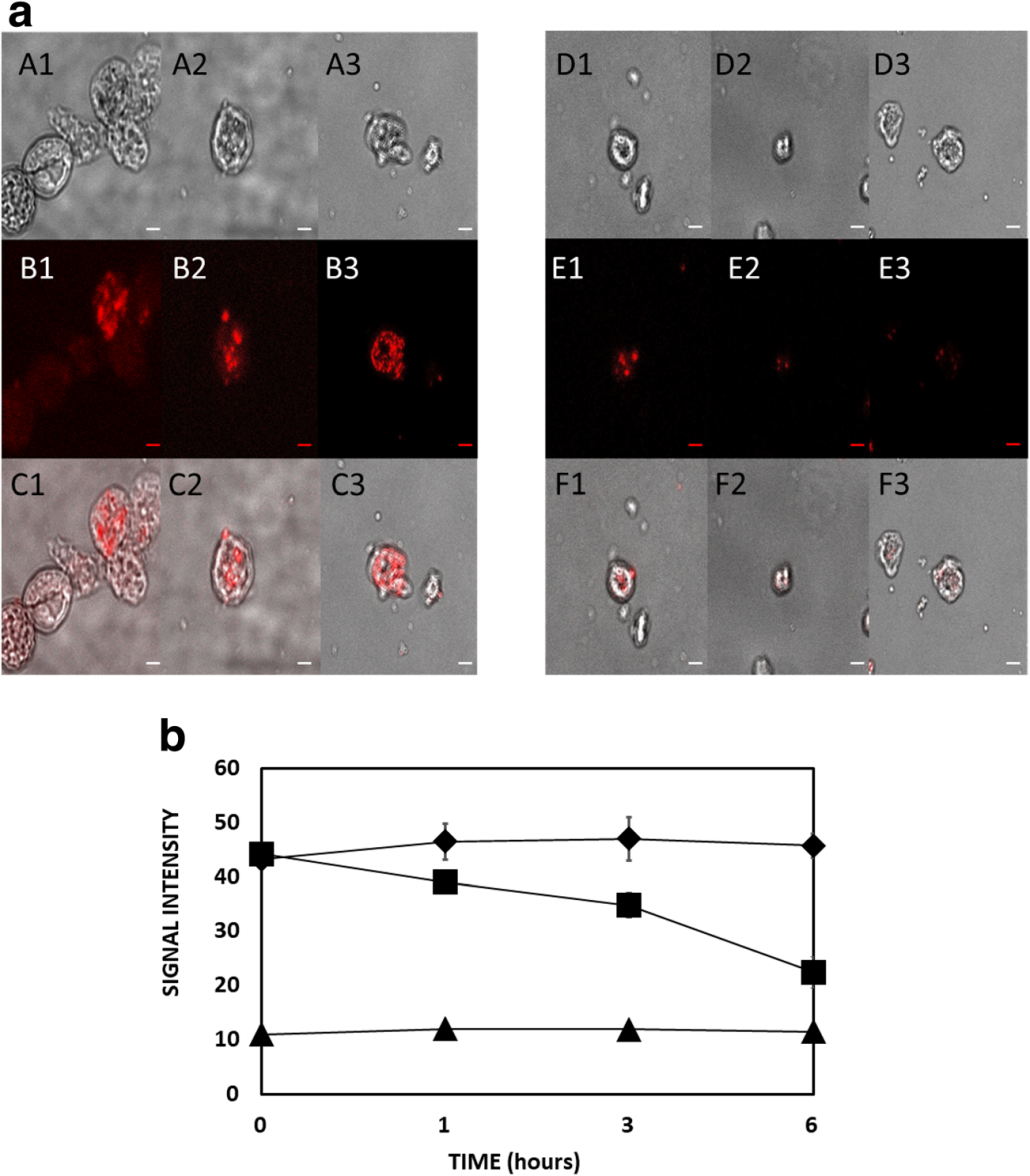
**A**. Respiration activity of control and IC_90_ G418 treated *Acanthamoeba* trophozoites as a function of time. Signal intensity of the CTC reporter indicates respiration activity. Left column represents fluorescence intensity of untreated trophozoites while the right column fluorescence intensity of G418-treated amoebae at 1, 3 and 6 h (A1, A2 and A3, B1, B2 and B3 and so on) after G418 induction. A1–A3 and D1–D3 phase contrast), B1–B3 and E1–E) formosan fluorescence, C1–C3 and F1–F3 phase and fluorescence combined. Images are representative of three independent experiments. Scale bar 5 μM. **B**. CTC formazan signal intensity from untreated (black diamond) and IC_90_ G418 treated (black square) *Acanthamoeba* trophozoites when excited at 350 nm, at 1, 3 and 6 h. *Acanthamoeba* with no CTC loaded (black up-pointing triangle)

The reduction of CTC to fluorescent formazan was used to assay the respiratory activity of mitochondria within *Acanthamoeba* trophozoites. Incubation of trophozoites with CTC resulted in the accumulation of fluorescence through the action of mitochondria. This fluorescence became progressively less intense in cells treated with G418 throughout the six-hour period over which it was monitored indicating an inhibition of mitochondrial respiration (Fig. 6a). No such reduction was seen in control amoebae populations. This gradual mitochondrial respiratory inhibition was quantified spectrophotometrically (Fig. 6b).

**Fig. 7:**
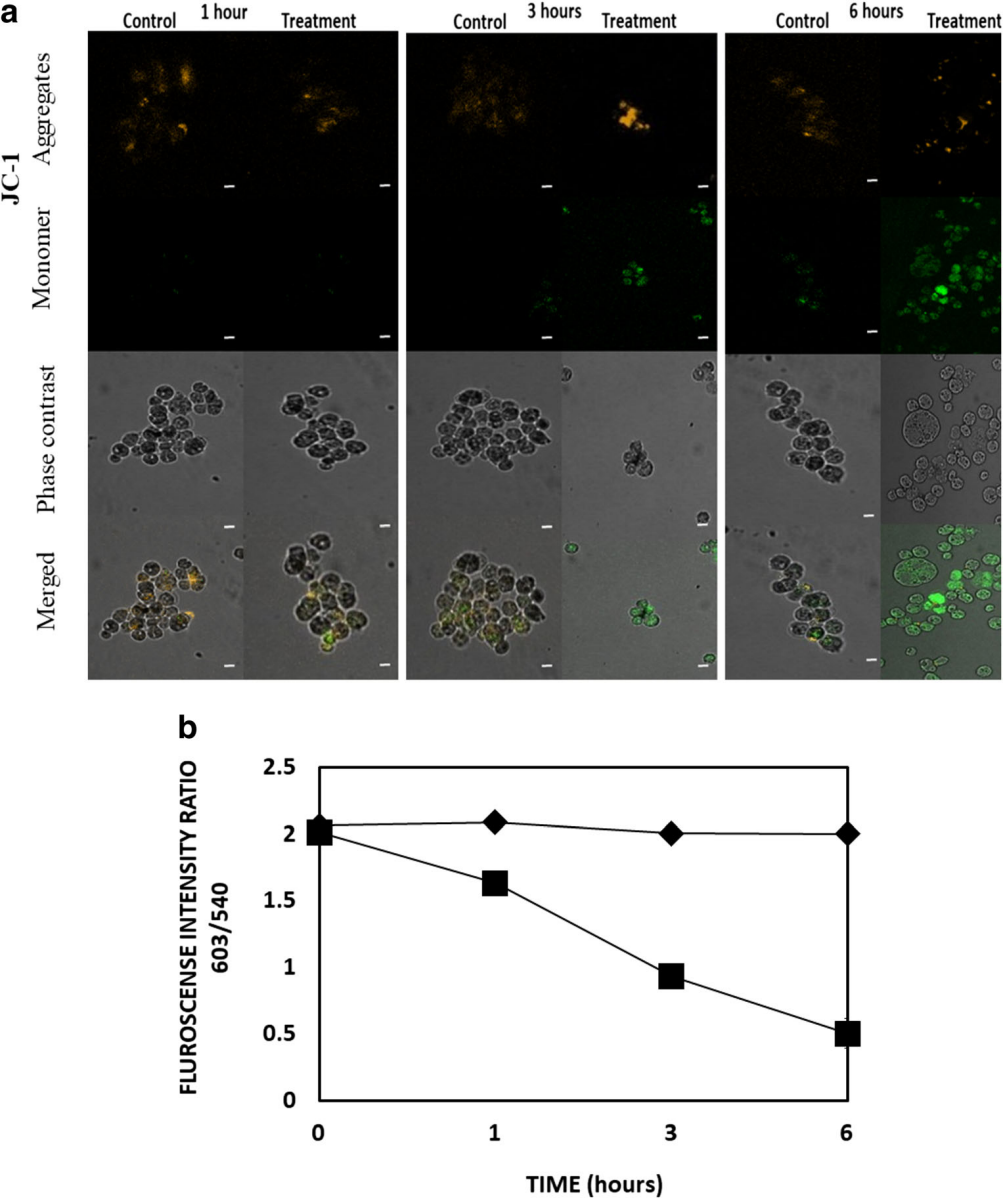
**A**. Fluorescence microscopy of JC-1 loaded, untreated and IC_90_ G418 treated *Acanthamoeba* trophozoites at 1, 3 and 6 h of incubation. Top row JC-1 aggregates (high ΔΨ_m_), second row JC-1 monomer fluorescence (low (ΔΨ_m_). Third row phase contrast. Fourth row phase contrast merged with JC-1 monomer fluorescence. Images are representative of three independent experiments. Scale bar 10 μM. **B**. JC-1 Bars represent fluorescence intensity ratio 603/540 of untreated (black diamond) and IC_90_ G418 treated (black square) *Acanthamoeba* trophozoites, which indicates ΔΨ_m_ alterations at 1, 3 and 6 h. Low ratio corresponds to depolarization while high hyperpolarization

**Fig. 8:**
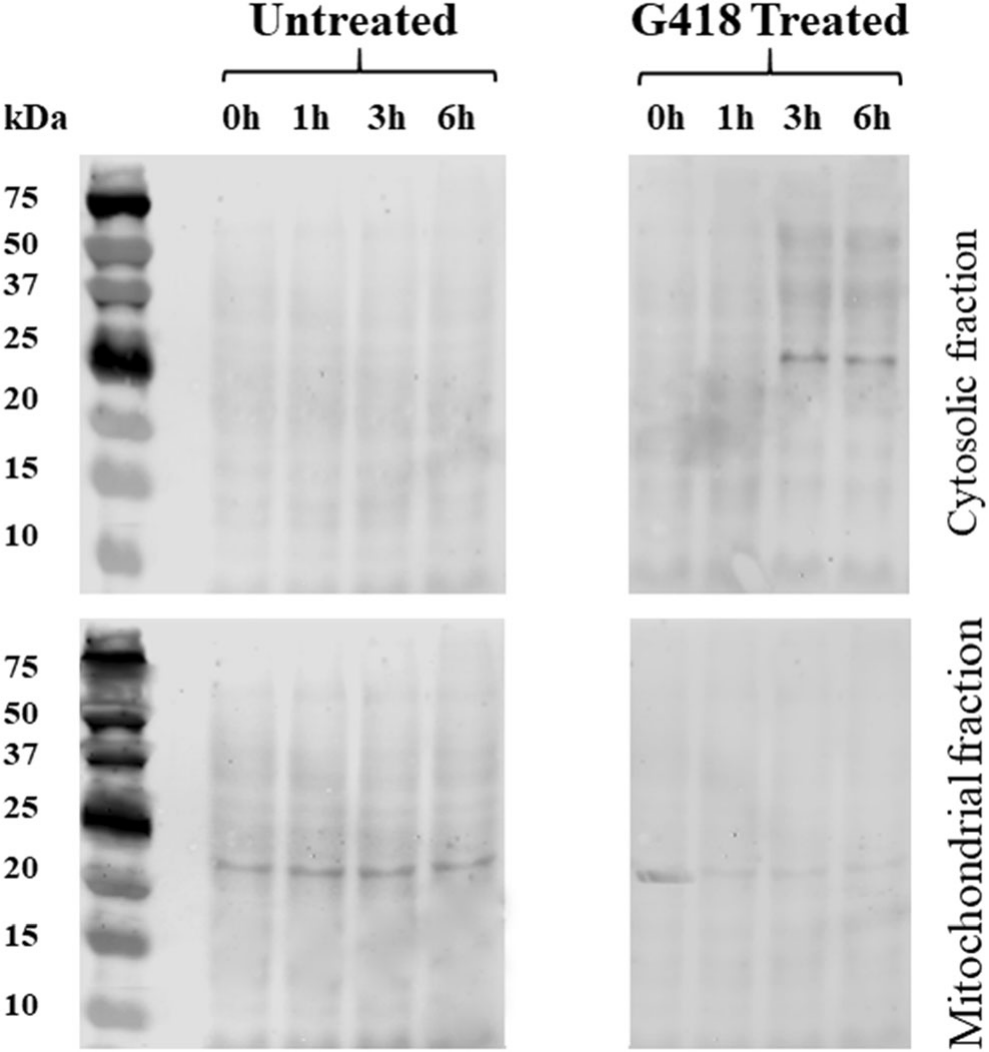
Upper panels show Western Blots from the cytosolic faction of *Acanthamoeba* stained with anti-cytochrome *c* antibody at four-time points. Lower panels show the same but for the mitochondrial fraction. Pre-stained molecular weight markers are shown in the left most lane of the panels

Analysis of the fluorescent intensities retrieved from JC-1 loaded trophozoites, revealed that mitochondrial dysfunction is based on changes occurring in mitochondrial membrane potential disturbance (ΔΨ_m_) rather than mitochondrial breakage. It was shown that JC-1 fluorescence ratio was stable in untreated trophozoites during a period of treatment, while at the same time the fluorescence ratio of the G418 treated *Acanthamoeba* has experienced a noticeable fall. The ratio is representative of 603/504 fluorescence intensities which in turn represents the relative amount of the dye that exist as monomer or aggregate. Analysis of the readings has shown a marked decrease in ΔΨ_m_ ranges from 24% at the first hour to 58% at 3 h and to 70% at 6 h (Fig. 7a). Fluorescent microscopy also revealed that J-aggregate fluorescence, which is marker of healthy and functional mitochondria, is decreased dramatically throughout G418 treatment (Fig. 7b). Western blotting of fractionated *Acanthamoeba* trophozoites showed that cytochrome *c* is released from mitochondria in cells treated with G418 (Fig. 8). After 3 h of G418 treatment, most of the cytochrome *c* was found in the cytosolic fraction.

## Discussion

It is well known that various types of programmed cell deaths are necessary part of embryogenesis and development in higher multicellular eukaryotes. For example, cells are required to die in the limb-bud to define digits in the developing limb (Zaleske 1985) and cells of the immune system that react to self must die during the process of thymic education (Murphy et al. 1990). Programmed cell death has also been described in many unicellular organisms including yeast (Madeo et al. 1999), several protozoans (Cornillon et al. 1994; Welburn et al. 1996; Olie et al. 1998; Verdi et al. 1999; Al-Olayan et al. 2002) and even bacteria (Yarmolinsky 1995). Whereas PCD makes sense in metazoans, it is less obvious why a single cell organism such as *Acanthamoeba* might express proteins to destroy itself. It is suggested that PCD may be in the interest of parasite populations within a host to limit the parasite load thereby allowing reproduction and spread of more parasites in the longer term (Al-Olayan et al. 2002; Nguewa et al. 2004), but in free living amoebae, PCD may be a mechanism to prevent the spread of pathogenic bacteria and viruses through local populations.

In agreement with former studies (Martín-Navarro et al. 2015; Martín-Navarro et al. 2017; Sifaoui et al. 2017; Baig et al. 2017; Lopez-Arencibia et al. 2017; Moon et al. 2018), we have found morphological changes in *Acanthamoeba* associated with a PCD-linked series of events. These include rounded up, cell shrinkage, intracellular ion fluctuations, mitochondrial dysfunction nuclear and chromatin condensation and finally breakup and release of apoptotic body-like particles. Confocal microscopy revealed chromatin condensation reflected by an increase in the intensity of Hoechst staining while the nucleus began to vesiculate. However, electrophoresis after DNA isolation of the G418 treated trophozoites did not reveal DNA cleavage or the ladder-like characteristic pattern seen in *Entamoeba* (Villalba et al. 2007).

An elevation of [Ca^2+^]_i_ has been associated with early and late stages of PCD in a wide variety of cell types and many PCD proteins are calcium sensitive (Orrenius et al. 2003). We have found that [Ca^2+^]_i_ also rises in *Acanthamoeba* in the presence of G418, before any sign of cellular death was apparent. A steady rise in [Ca^2+^]_i_ was observed in the presence of 75 μg/mL G418 in treated *Acanthamoeba* trophozoites from a resting [Ca^2+^]_i_ level around 20 nM rising to 60 nM plateau around 90 min. This is similar to the rise in [Ca^2+^]_i_ of *Entamoeba histolytica* caused by G418 (10 μg/mL) in (Villalba et al. 2007) where resting levels were 20 nM and increased to around 47 nM after 120 min. The PCD inducer of *Dictyostelium*, DIF also caused an increase in [Ca^2+^]_i_ (Schaap et al. 1996).

It is reported that the mitochondria of *A. castellanii* actively accumulate Ca^2+^ which leads to a decrease in mitochondrial membrane potential ∆Ψ_m_ (Domka-Popek and Michejda 1986; Trocha and Stobienia 2007). We have found that mitochondrial function as measured by CTC and JC-1 decreased as the [Ca^2+^]_i_ increased. Furthermore, we have also discovered the release of cytochrome *c* from *Acanthamoeba* mitochondria by Western Blot analysis (Fig. 8). The well-characterised antibody reacted to a band at around 25 kDa, close to double that of the 12.9 kDa size expected from the encoding sequence of the conserved *Acanthamoeba* cytochrome *c* gene; however, the *Acanthamoeba* protein contains an exposed cysteine residue at position 1213 (see red arrow in Supplementary fig. 2) and as it is known that cytochrome *c* has a strong propensity to polymerise through domain-pair exchange (Hirota et al. 2010), requiring denaturation conditions to convert to the monomeric state (Margoliash and Lustgarten 1962). We hypothesise that the 25 kDa band that is recognised by the anti-cytochrome *c* antibody is a homodimer (possibly stabilized/mediated by cysteine disulphide bonds) of *Acanthamoeba* cytochrome *c*. Spectrometric analysis of mitochondria from stressed *Acanthamoeba* also indicate that cytochrome *c* is released (Trocha and Stobienia 2007). Cytochrome *c* release is known to be calcium-dependent in vertebrate neurons (Schild et al. 2001) and cytochrome *c* released from mitochondria is known to accumulate in the nucleus where it is implicated in chromatin remodelling (Nur-E-Kamal et al. 2004). It is interesting to note that the peroxidase activity of dimeric cytochrome *c* is far greater than the monomer perhaps making it a more potent mediator of PCD (Wang et al. 2011).

Several differences in G418 induced PCD compared to other inducers are evident. When compared directly, different amoebicidal drugs show differences in PCD profile (Moon et al. 2018) perhaps indicating that they are activating different pathways or different targets in a single pathway. In summary, we have found that G418 causes PCD-like changes in *Acanthamoeba*. In the early stages of the process, there is an increase in intracellular calcium concentration leading to mitochondrial misfunction and the release of cytochrome *c*.

The biological pathways that lead to PCD in *Acanthamoeba* are far from being fully understood; however, an understanding of cell death-triggering mechanisms, mediators, and executioner pathways will offer the potential to identify targets for therapy, not only for *Acanthamoeba* infections but for many other disease-causing protists that share these PCD pathways.

## Electronic supplementary material

Supplementary Figure 1 Maximum likelihood tree of 18S ribosomal gene fragments showing that our fresh isolate GS-336 is closely related to the well characterised Neff strain of *Acanthamoeba* within the type T4 group. Branch supports are given at the nodes where they are greater than 0.4. (PNG 835 kb)

High-resolution image (TIF 120 kb)

Supplementary Figure 2 A lineup of cytochrome *c* proteins from various species with *Acanthamoeba* at the top. The peptide used to raise the antibody used is highlighted showing that this is highly conserved. Note also the cysteine residue at position 13 in the *Acanthamoeba* sequence which is unusual (see red arrow). The blots (Fig. 8) show a band at around 25 kDa rather than the expected 14 kDa but cytochrome *c* is known to polymerise (Hirota et al. 2010) and so it is likely that the epitope recognised by the antibody is a cytochrome *c* dimer. (PNG 2122 kb)

High-resolution image (TIF 477 kb)
