# Supplementary figures and images for: G418 induces programmed cell death in Acanthamoeba through the elevation of intracellular calcium and cytochrome c translocation
Source: Parasitol Res. 2019 Jan 7;118(2):641–51. doi: 10.1007/s00436-018-6192-0 (PMC6349814; doi:10.1007/s00436-018-6192-0)

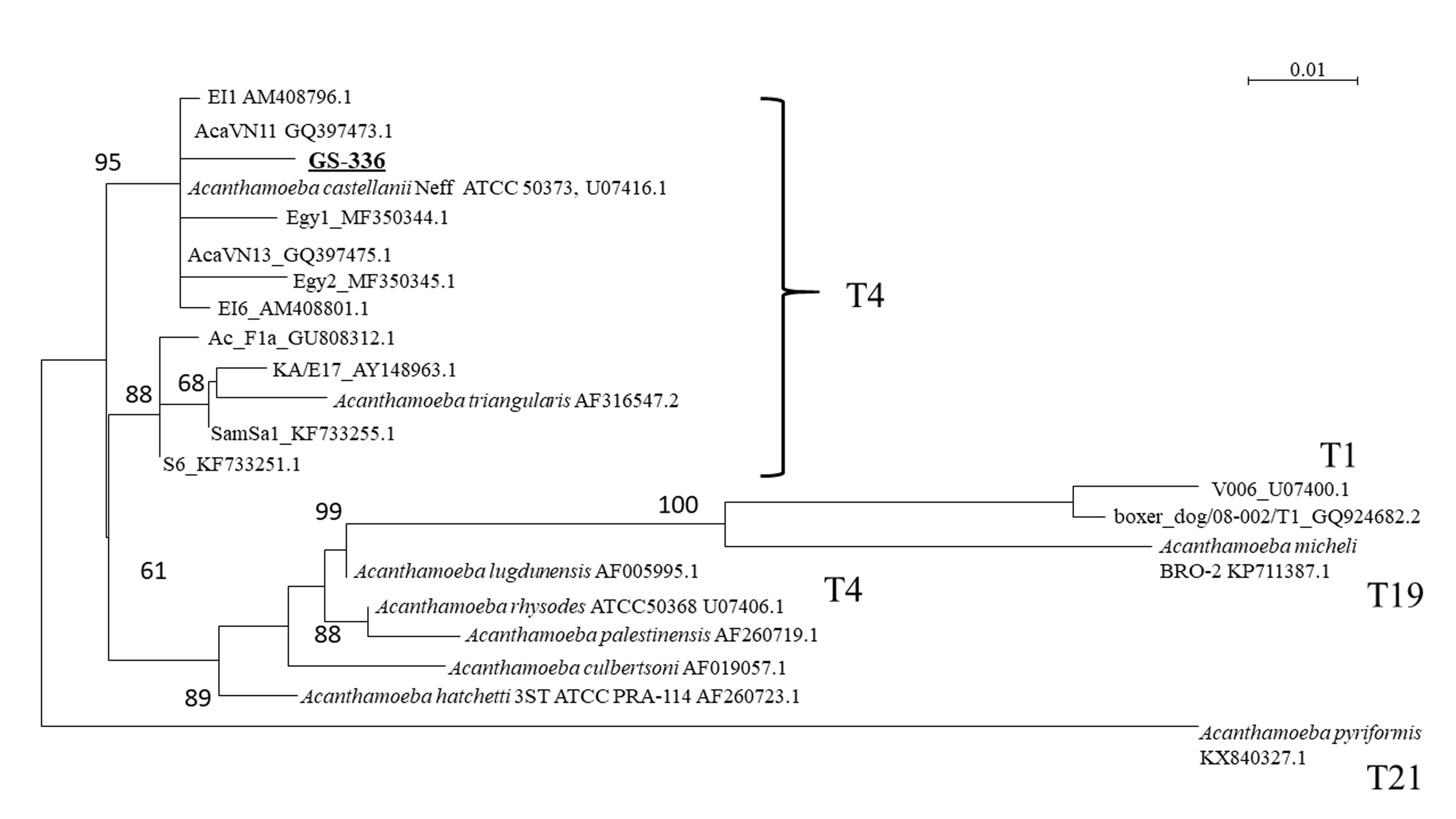

Supplement: Supplementary file 1 — Maximum likelihood tree of 18S ribosomal gene fragments showing that our fresh isolate GS-336 is closely related to the well characterised Neff strain of Acanthamoeba within the type T4 group. Branch supports are given at the nodes where they are greater than 0.4. (PNG 835 kb) [file 436_2018_6192_Fig9_ESM.png]

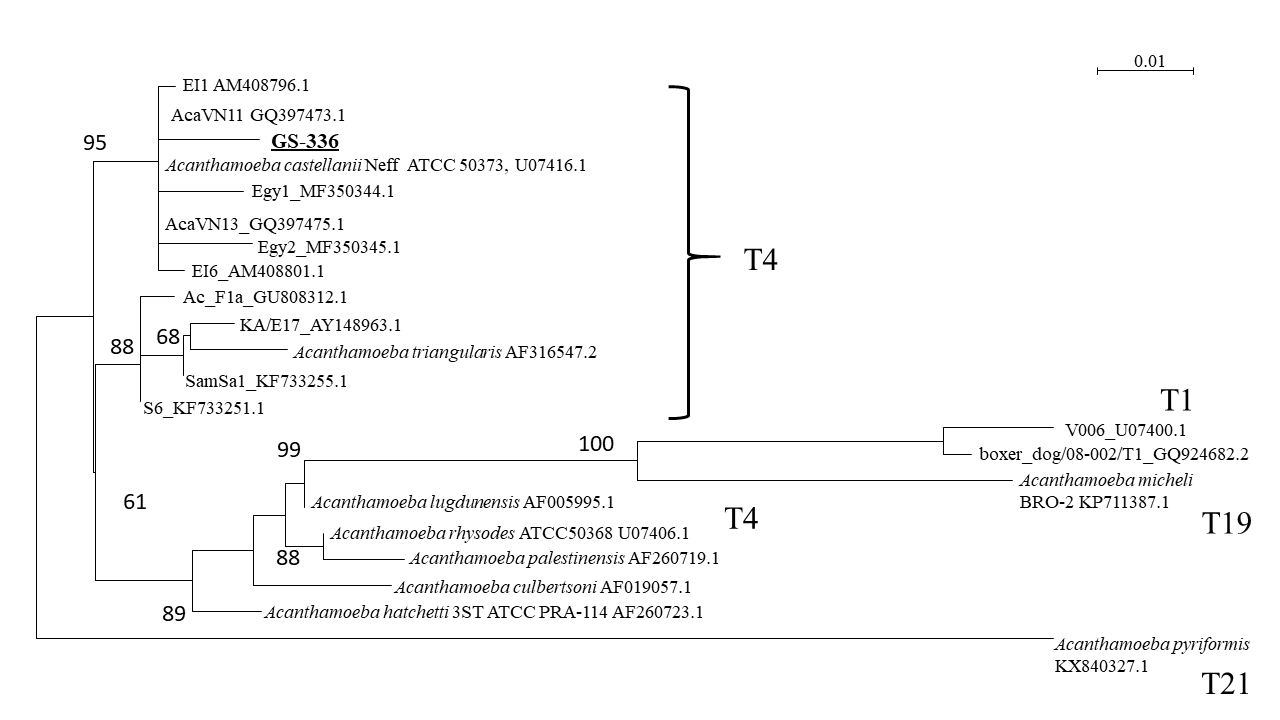

Supplement: Supplementary file 2 — High-resolution image (TIF 120 kb) [file 436_2018_6192_MOESM1_ESM.tif]

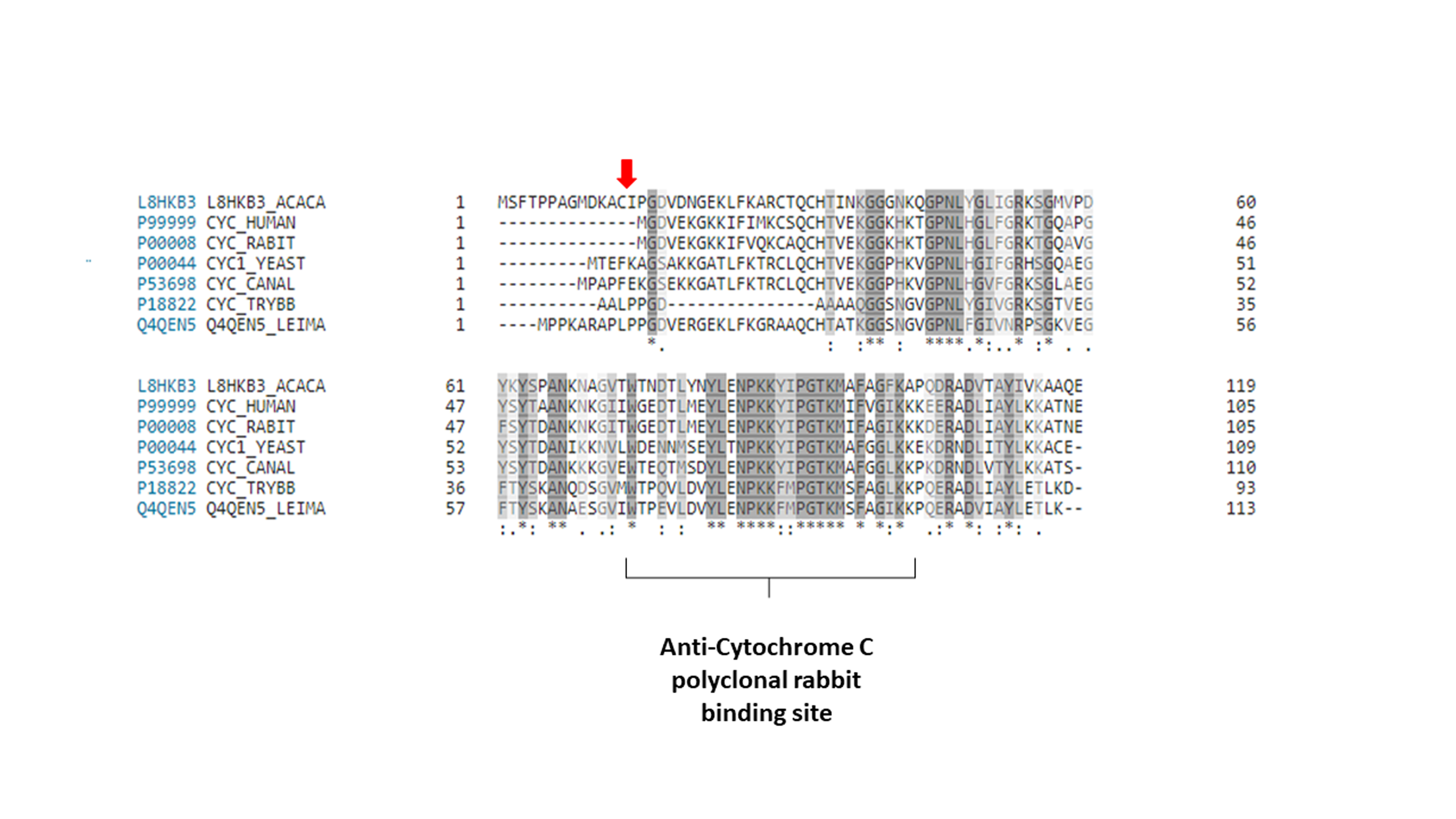

Supplement: Supplementary file 3 — A lineup of cytochrome c proteins from various species with Acanthamoeba at the top. The peptide used to raise the antibody used is highlighted showing that this is highly conserved. Note also the cysteine residue at position 13 in the Acanthamoeba sequence which is unusual (see red arrow). The blots (Fig. 8) show a band at around 25 kDa rather than the expected 14 kDa but cytochrome c is known to polymerise (Hirota et al. 2010) and so it is likely that the epitope recognised by the antibody is a cytochrome c dimer. (PNG 2122 kb) [file 436_2018_6192_Fig10_ESM.png]

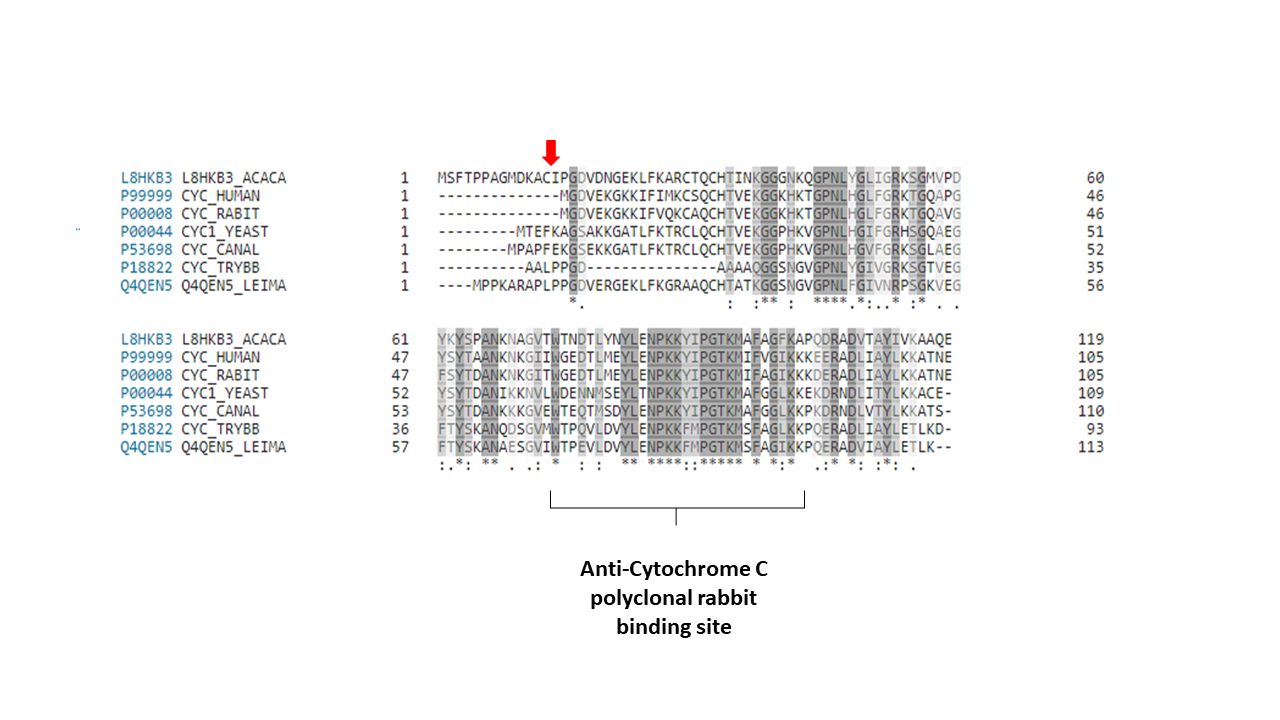

Supplement: Supplementary file 4 — High-resolution image (TIF 477 kb) [file 436_2018_6192_MOESM2_ESM.tif]
